# Virtual Reality Exposure Therapy for Reducing School Anxiety in Adolescents: Pilot Study

**DOI:** 10.2196/56235

**Published:** 2024-11-05

**Authors:** Gesa Beele, Paula Liesong, Sabine Bojanowski, Kristian Hildebrand, Malte Weingart, Julia Asbrand, Christoph U Correll, Nexhmedin Morina, Peter J Uhlhaas

**Affiliations:** 1 Department of Child and Adolescent Psychiatry Charité-Universitätsmedizin Berlin Berlin Germany; 2 Berlin University of Applied Sciences and Technology Berlin Germany; 3 Department of Clinical Psychology for Childhood and Adolescence University of Jena Jena Germany; 4 Department of Psychiatry and Molecular Medicine Donald and Barbara Zucker School of Medicine at Hostra/Northwell Hempstead, NY United States; 5 Department of Psychiatry The Zucker Hillside Hospital, Northwell Health Glen Oaks, NY United States; 6 Institute of Psychology University of Münster Münster Germany; 7 Institute of Neuroscience and Psychology University of Glasgow Glasgow United Kingdom

**Keywords:** virtual reality exposure therapy, VRET, school anxiety, social anxiety, adolescents, virtual reality, VR, autonomic arousal, exposure therapy, posttreatment, digital health, simulation

## Abstract

**Background:**

Virtual reality exposure therapy (VRET) is a promising treatment approach for anxiety disorders. However, while its efficacy has been demonstrated in adults, research on the efficacy of VRET in the treatment of adolescents with anxiety disorders is largely lacking.

**Objective:**

A pilot study was carried out to test whether exposure to a virtual reality (VR) school environment elicits state anxiety and autonomic arousal in adolescents with school anxiety (diagnoses covering social anxiety disorder or specific phobia involving school contexts). In addition, we examined whether repeated VR exposure led to a reduction in this fear response, trait school anxiety, and social anxiety symptoms. Moreover, the relationship of presence, the subjective sense of “being there,” during VR exposure with anxiety measures and treatment response was examined.

**Methods:**

In a pilot study, 10 adolescents with school anxiety (age range 14 to 17 years) participated in five VRET sessions. Self-reported state anxiety, heart rate, and presence during exposure, as well as trait school anxiety and social anxiety before and after treatment, were measured.

**Results:**

The VR scenario induced state anxiety and autonomic arousal. After VRET, a significant reduction in state anxiety (η^2^=0.74) and social anxiety symptoms (*d*=0.82) as well as a trend toward a decrease in trait school anxiety were observed, while autonomic arousal did not change. In addition, presence during VR exposure was associated with state anxiety and treatment response.

**Conclusions:**

Our findings indicate the feasibility and potential effectiveness of VRET as a treatment method for symptoms of school and social anxiety in adolescents.

## Introduction

Anxiety disorders are the most common mental disorders in adolescence, with approximately 25% of adolescents meeting diagnostic criteria within 12 months [1]. Anxiety disorders frequently manifest in educational contexts [2-4]. School anxiety is a syndrome that summarizes fears of school-related situations such as fear of failure in performance situations, or fear of social situations [5,6]. It repeatedly leads to avoidance behavior including school refusal [7-9]. Prevalence for anxiety-based school refusal ranges from 1% to 4% of students over a 12-month period [7,10], although these figures must be interpreted cautiously due to differing conceptualizations and limited research [11]. School anxiety is not classified as a disorder in diagnostic manuals [12,13]. However, school anxiety and absenteeism are associated with an increased risk of poor academic performance and impaired social functioning [6,14-17].

School anxiety can occur within different anxiety disorders, such as specific phobias involving school contexts, and particularly in social anxiety disorder (SAD) [6,18]. These disorders overlap with school anxiety in terms of fears, symptoms, and consequences [5,11,19]. SAD entails anxiety in different social interaction and performance situations, most often including the school setting [20,21].

The most common intervention for school anxiety, SAD, and specific phobias is cognitive behavioral therapy (CBT), which has been demonstrated to promote attendance at school and reduction of anxiety symptoms [11,22-25]. An important component of CBT is exposure in vivo, which is associated with large effect sizes in the treatment of anxiety disorders [26,27]. However, despite its effectiveness, exposure in vivo is not consistently used for children and adolescents with anxiety disorders [28,29]. Reasons include lack of resources and inconvenient scheduling of sessions [30,31], especially in school settings. In addition, exposure to school-related stimuli requires a high level of cooperation with the school [32].

Virtual reality exposure therapy (VRET) may overcome some of these challenges. VRET involves exposure to feared objects and situations using interactive virtual environments via head-mounted displays and headphones, thus enabling greater control and an individual adaptation of the exposure situation [33]. VRET has been rated by therapists to be more practical in the treatment of SAD than exposure in vivo [34]. Moreover, youth who are unwilling to participate in exposure in vivo may be more likely to choose VRET because it could be more acceptable [35,36].

Several studies have provided evidence for the efficacy of VRET in adults with anxiety disorders, showing comparable effect sizes in symptom reduction as in vivo exposure [33,36]. The effects appear to generalize to daily life and are maintained [37-39]. Although specific phobias have been most frequently studied to date, a growing body of research also shows promising results for SAD, reporting reductions in state and trait anxiety [34,40-42]. However, evidence for the effectiveness of VRET in the treatment of children and adolescents is largely lacking [43].

Preliminary research suggests that VRET is acceptable to adolescents and is associated with reductions in anxiety symptoms in youth (eg, [43-46]). Kahlon et al [47] reported a reduction in public speaking anxiety in adolescents after a speech task in a virtual classroom which was maintained at follow-up. To date, only one study examined VRET for school anxiety [48], indicating reductions in school-related fears. However, this study recruited participants in schools without a formal clinical diagnosis and combined VRET with other methods such as relaxation training.

The principles underlying VRET are not yet fully understood. However, it is assumed that the working mechanisms of VRET mirror those of exposure in vivo [49]. Accordingly, previous accounts of VRET refer to the “emotional processing theory” [50] that proposes that repeated confrontation with anxiety-relevant stimuli gradually reduces emotional and physiological reactivity by altering the cognitive representation of these stimuli. The “inhibitory learning model” [51] emphasizes the relevance of expectancy violations, that is, the mismatch between expected and actual outcomes during exposure. It postulates that through repeated exposure to anxiety-relevant stimuli, patients learn that the feared consequences do not occur or are less severe than expected. However, as many feared outcomes cannot occur in VRET, and therefore, cannot be tested, the effectiveness of VRET can probably not be solely attributed to the violation of expectancies [49,52,53]. Therefore, it is suggested that multiple mechanisms may contribute to anxiety reduction in VRET [49]. The reduction of fear (habituation) and expectancy violations will often occur in an equivalent manner and only slightly influence the design of the actual exposure session [54].

One important factor regarding the mechanisms of VRET itself might be presence, which refers to the subjective interpretation of the virtual environment as if it were real, feeling engaged, and connected to it [55,56]. Presence is considered a precondition for evoking anxiety through virtual scenarios, as it indicates that the virtual environment feels realistic and attracts the focus of attention [57-59]. It may therefore also be relevant for therapeutic efficacy [55]. However, the findings regarding the relationship between presence and treatment outcome are mixed [47,55,59,60].

This study examined VRET to reduce school anxiety in adolescents with a diagnosis of SAD or specific phobia involving school contexts. We hypothesized that a virtual school scenario would elicit self-reported state anxiety and autonomic arousal (heart rate [HR]) in adolescents with school anxiety. Moreover, we expected state anxiety, autonomic arousal, and trait school, as well as social anxiety, would be reduced after VRET. Moreover, we assumed that there is a positive association between a sense of presence during the virtual reality (VR) sessions and anxiety measures, as well as treatment response.

## Methods

### Participants

A total of 13 adolescents aged 12 to 18 years were recruited from in and outpatient services from the Clinic for Psychiatry, Psychosomatics and Psychotherapy of Children and Adolescence, Charité Universitätsmedizin Berlin. Inclusion criteria were (1) school anxiety assessed by the current psychotherapist and (2) a diagnosis of SAD (F40.1) or specific phobia involving school contexts (F40.2) according to the *ICD-10* (*International Statistical Classification of Diseases, Tenth Revision*) [13]. Exclusion criteria were acute suicidality, motion sickness, and visual impairments.

Three participants did not complete the assessments due to a suicide attempt not related to the study (n=1), a delay caused by coronavirus disease, and one participant did not wish to complete the study. The sample characteristics (n=10) are depicted in Table 1. All participants had a diagnosis of SAD.

**Table 1:** Baseline demographic and clinical characteristics.

Characteristics				Value (N=10)
**Age**				
	Mean (SD)			15.7 (0.9)
	Minimum			14
	Maximum			17
**Class**				
	Mean (SD)			10
	Minimum			9
	Maximum			11
**School anxiety^a^**				
	**Test anxiety**			
		Mean (SD)	12.9 (2.4)	
		Minimum	15	
		Maximum	8	
	**General anxiety**			
		Mean (SD)	12.5 (1.7)	
		Minimum	15	
		Maximum	9	
	**School reluctance**			
		Mean (SD)	7.9 (1.6)	
		Minimum	10	
		Maximum	5	
**Social anxiety^b^**				
	Mean (SD)			17.7 (2.3)
	Minimum			13
	Maximum			21
**Relative frequency, n (%)**				
	Sex (female)			6 (60)
	School type (grammar school)			7 (70)
	Diagnosis (SAD^c^)			10 (100)
	Comorbid disorders^d^			9 (90)

^a^School anxiety was measured with the Anxiety Questionnaire for Pupils.

^b^Social anxiety was measured with the Diagnostic System for Mental Disorders in Children and Adolescents in German (DISYPS-III-SBB-ANG).

^c^SAD: social anxiety disorder (F40.1).

^d^Comorbid disorders: F32.1/2, F40.2, F41.0, F42.2, F50.0/8, F81.0.

### Ethical Considerations

The study was approved by the ethics committee of the Charité Universitätsmedizin (EA2/254/21). Both written and oral informed consent was given by participants and their caregivers. All participants received an expense allowance of 10 € (approximately US $11) per hour for their participation in the study.

### Psychological Assessments

To examine school-related trait anxiety, the Angstfragebogen für Schüler (AFS; English: Anxiety Questionnaire for Pupils) [61] was used*.* This self-report questionnaire consists of 50 items, which are to be answered on a dichotomous scale (correct, not correct), that are categorized into four subscales: (1) test anxiety: fear of exam situations or performance failure (eg, “I am always afraid that I will get bad grades during exams”), (2) general (manifest) anxiety: general anxiety symptoms and reduced self-confidence (eg, “I worry too much”), (3) school reluctance: inner resistance and loss of motivation toward educational matters (eg, “I am often bad-tempered in class”), and (4) social desirability which assesses the response behavior of the participants (eg, “I have never lied”). The latter subscale was not included in this study, as it does not measure school anxiety and was therefore not considered relevant for the purpose of the study. The internal consistencies of the reported scales (α=.73 to α=.89) and the test-retest reliability after one month (rtt=0.71 to rtt=0.76) are high. Validity and sensitivity to change were supported by correlations with construct-related measures and various studies [61].

The anxiety disorders section of the Diagnostic System for Mental Disorders in Children and Adolescents in German (DISYPS-III-SBB-ANG) [62] was used for the assessment of social anxiety symptoms. Responses range from 0 (not at all) to 3 (particularly). The authors have reported a good internal consistency of the subscale (α=.87) but not test-retest reliability. The validity of the scale was supported by examinations of convergent and divergent validity [62].

In addition, the Subjective Units of Distress (SUD) [63] was used as a measure of subjective state anxiety. It was used as a verbal self-report during each phase of the scenario (“On a scale from 0 (relaxed or no fear) to 10 (biggest fear ever), how high is your fear right now?”). Correlations with autonomic stress parameters [64] and an established state anxiety questionnaire [65,66] support the validity of the SUD.

To assess the presence in virtual environments, the Igroup Presence Questionnaire (IPQ) [56] was administered. The IPQ is a self-report questionnaire that includes 14 items which are rated on a 7-point Likert scale ranging from 0 (not at all) to 6 (very much). It comprises three subscales (spatial presence, involvement, and experienced realism) and one general item about global presence. The IPQ has demonstrated good psychometric properties with a high internal consistency of the total score (α=.85 to α=.87) [56,67].

### Autonomic Measures

Autonomic parameters were continuously assessed throughout the sessions using the eSense Pulse system (Mindfield Biosystems), consisting of a chest belt containing a 1-channel-electrocardiogram with 500 Hz sampling. After the measurement, the autonomic data were divided according to the different phases of the VR sessions. For inspecting and preprocessing the interbeat intervals data, the program ARTiiFACT [68] was used, with artifacts detected by absolute median deviation, manually checked, and replaced by cubic spline interpolation. Mean HR, influenced by the sympathetic and parasympathetic nervous system, was calculated for each phase of the first and the final session.

### Virtual Reality Equipment

Participants wore a head-mounted display (VR glasses) and headphones (VIVE Pro). Participants further used two controllers for walking and jumping, as well as grabbing and carrying objects. The VR scenario was implemented using the VR simulation software Game Engine Unity 2020 (Unity Technologies).

### Procedure

Participants completed 5 largely standardized exposure sessions within a one-month period, which were conducted by two graduate master-level students of clinical psychology in an office at the Department of Child and Adolescent Psychiatry, Charité (for study protocols, see Multimedia Appendix 1). Before the first and after the final session, participants completed the AFS and DISYPS. The first session began with psychoeducation about the rationale of exposure and the relevance of safety and avoidance behaviors. In the following sessions, the rationale was repeated and the adolescents’ negative expectations of potential outcomes in the exposure were verbally assessed. During exposure, safety and avoidance behaviors were prevented when distraction or avoidance were observed. The HR was continuously measured.

**Figure 1:**
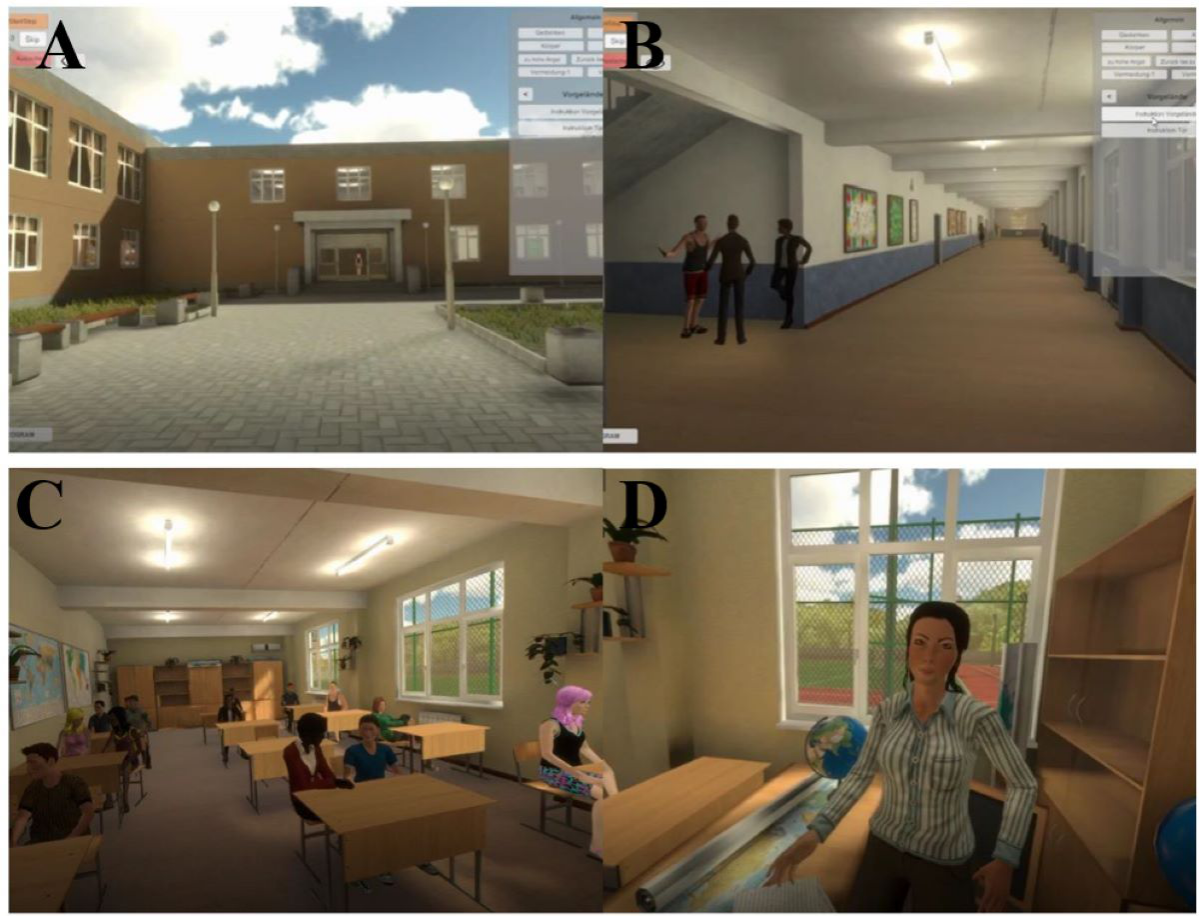
Virtual reality scenario. The upper pictures are from the experimenter’s perspective, and the lower pictures are from the participants’ perspective. Panels (A; schoolyard) and (B; school corridor) are examples of the phase “exploration.” The lower pictures are related to the phases “introduction,” “math,” and “event.”.

The VR scenario (Figure 1) consisted of the following phases which were controlled by the experimenter: (1) at a street in front of the school grounds, participants started to get used to the VR (“acclimatization”). (2) They could freely explore the schoolyard and school building (“exploration”). (3) Participants went to the classroom and sat at a table while typical school sounds were presented (“class”). (4) The ringing of a school bell announced the start of class and the participants were asked by the teacher to come to the front of the class and complete three tasks which were motivated by the Trier Social Stress Test [69,70]: (a) introducing themselves (“introduction”), (b) solve small arithmetic problems verbally (“math”), and (c) talk about a recent personal experience (“event”). Through prerecorded sentences, questions, and comments (in a neutral tone), the virtual teacher could interact with the participants. From the second session onwards, the virtual classmates responded to the participants by looking at them, laughing, or applauding. During each phase, the participants were asked to rate their SUD. From the phase “event” onwards, the assessment was repeated at regular intervals and a mean value was calculated afterward. Finally, the 3 tasks (“introduction,” “math,” and “event”) were repeated in a randomized order until self-reported state anxiety had decreased by about 40% [71]. Following these tasks, the participants sat down (“end”) until the end of the session.

Following each VR session, the experimenter discussed with the adolescents the occurrence of their negative expectations. Finally, participants completed the IPQ. Each session lasted about 60 minutes in total.

### Statistical Analysis

Statistical analyses were performed using RStudio (version 2023.06.1; Posit PBC). SUD and HR as dependent variables within and between sessions were each analyzed using two-factor repeated measures ANOVAs (rmANOVA), with repeated measures factors session (first, final) and phase (“acclimatization,” “exploration,” “class,” “introduction,” “math,” “event,” “end”). Pre- and postreductions in trait school anxiety and social anxiety symptoms as dependent variables were analyzed using 1-tailed, paired-samples *t* tests of the AFS subscales and the DISYPS’ social anxiety symptoms. Reductions in trait school anxiety were assessed for clinical significance with the reliable change index (RCI) [72], which was calculated using the SD and the test-retest reliability of the subscales from the normative sample of the AFS [61]. Clinical relevance was defined as the response criterion of a symptom reduction of 30% [73]. The 1-tailed Pearson product-moment correlations were calculated to examine the relationships between presence and state anxiety (IPQ total score and mean SUD and HR during the first session), as well as treatment response (mean IPQ total score across the five sessions and the post- and predifference scores of the AFS subscales).

Both uncorrected and corrected *P* values are reported. If the normal distribution of the data could not be confirmed with the Shapiro-Wilk test, nonparametric alternatives are reported. For rmANOVAs, Greenhouse-Geisser corrections were applied for variables that failed Mauchly’s test of sphericity.

## Results

### State Anxiety and Autonomic Arousal

**Figure 2:**
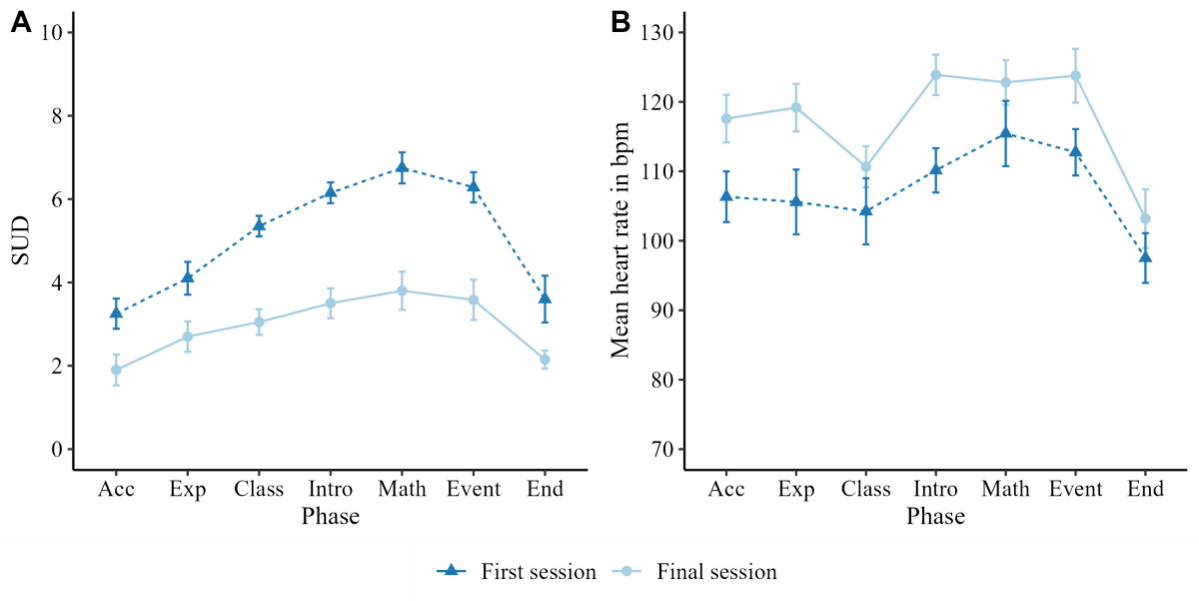
Mean trajectory of SUD (A) and HR (B) in the first and the final session. Note that n=10 for SUD and n=4 for HR. Acc: acclimatization; Exp: exploration; HR: heart rate; Intro: introduction; SUD: Subjective Units of Distress (state anxiety); Means and standard errors.

The rmANOVA for SUD (Table 2) resulted in a significant main effect of phase. Post hoc analyses (Multimedia Appendix 2) revealed that SUDs during the tasks inside the class, “class,” “introduction,” “math,” and “event” were significantly higher than during “acclimatization” and “end” (*t* values 4.3; *P* values .001; *P*s_corr._ .02. The main effect of the session and the session × phase interaction were also significant. Post hoc analyses showed that SUD was lower in all phases of the final session than the first session (*t* values 2.32; *P* values .046, *P*s_corr._ .046). In addition, there was a steeper increase and decrease in SUD in the first session than in the final session (Figure 2A).

**Table 2:** Results of repeated measures ANOVAs for SUD^a^ and autonomic arousal (HR^b^) in the first and the final session.

Variable and effect		*F* test (*df*)^c^	*P* value	Partial η^2^
**SUD (n=10)**				
	Phase	15.21 (6, 54)	<.001	0.63
	Session	25.49 (1, 9)	<.001	0.74
	Session × phase	4.56 (6, 54)	.006	0.34
**HR (n=4)**				
	Phase	10.84 (6, 18)	.03	0.78
	Session	3.00 (1, 3)	.18	0.50
	Session × phase	1.71 (6, 18)	.25	0.36

^a^SUD: Subjective Units of Distress (state anxiety).

^b^HR: heart rate.

^c^All *df*s for phase and session × phase were Greenhouse-Geisser-adjusted.

Due to technical connection problems between the pulse sensor and the PC, complete autonomic measurements were only available from 4 participants. The rmANOVA of HR revealed a significant main effect of phase (Table 2 and Figure 2B). Post hoc analyses (Multimedia Appendix 2) showed that HR was significantly higher during “introduction” and “math” than during “acclimatization,” “exploration,” and “end” (*t* values 3.76; *P* values .007; *P*s_corr_. .09). Neither the main effect of the session nor the session × phase interaction was significant, however. The participants’ individual trajectories corresponded with the reported effects (Multimedia Appendix 3).

### Trait School Anxiety and Social Anxiety

For trait school anxiety (AFS), the analyses revealed a significant reduction from pre to post-VRET in the subscale school reluctance and a trend toward significance in test anxiety, but not in general anxiety (Table 3). For social anxiety (DISYPS), the 1-tailed paired samples *t* test revealed a significant reduction from pre to post VRET (Table 3).

**Table 3:** Reductions in trait school anxiety and social anxiety from pre to post-VRET^a^.

Variable		Pre	Post	Test statistic^b^	*P* value	*P*_corr._^c^	ES^d^
**AFS^e^: trait school anxiety**							
	Test anxiety, mean (SD)	12.9 (2.4)	12.2 (2.8)	*t*_9_=–1.56	.08	.15	*d*=–0.26
	General anxiety, median (IQR)	12.5 (12.0-13.75)	12.0 (9.5-12.75)	V=4	.10	.15	*r*=0.49
	School reluctance, mean (SD)	7.9 (1.6)	7.0 (2.6)	*t*_9_=–1.87	.05	.14	*d*=–0.34
DISYPS^f^: trait social anxiety, mean (SD)		17.7 (2.3)	15.5 (3.0)	*t*_9_=–2.66	.01	–^g^	*d*=–0.82

^a^VRET: virtual reality exposure therapy.

^b^Analyses were 1-tailed paired-samples *t* tests except for general anxiety, for which the Wilcoxon signed rank test was used.

^c^Bonferroni-Holm correction was applied for corrected *P* values.

^d^ES: effect size.

^e^AFS: Anxiety Questionnaire for Pupils.

^f^DISYPS: Diagnostic System for Mental Disorders According to ICD-10 (International Statistical Classification of Diseases, Tenth Revision) and DSM-5 (Diagnostic and Statistical Manual of Mental Disorders [Fifth Edition]) in Children and Adolescents.

^g^Not applicable.

Regarding the clinical significance of the change in trait school anxiety, 7 of 10 participants did not show clinically significant improvements. One participant showed clinically significant improvements on the subscales general anxiety and school reluctance. Two participants showed a reduction >30% on at least one subscale, but no reliable change (RCI<|1.96|). The individual percentage change and the RCI of all participants can be found in Multimedia Appendix 4.

### Presence and Anxiety

**Figure 3:**
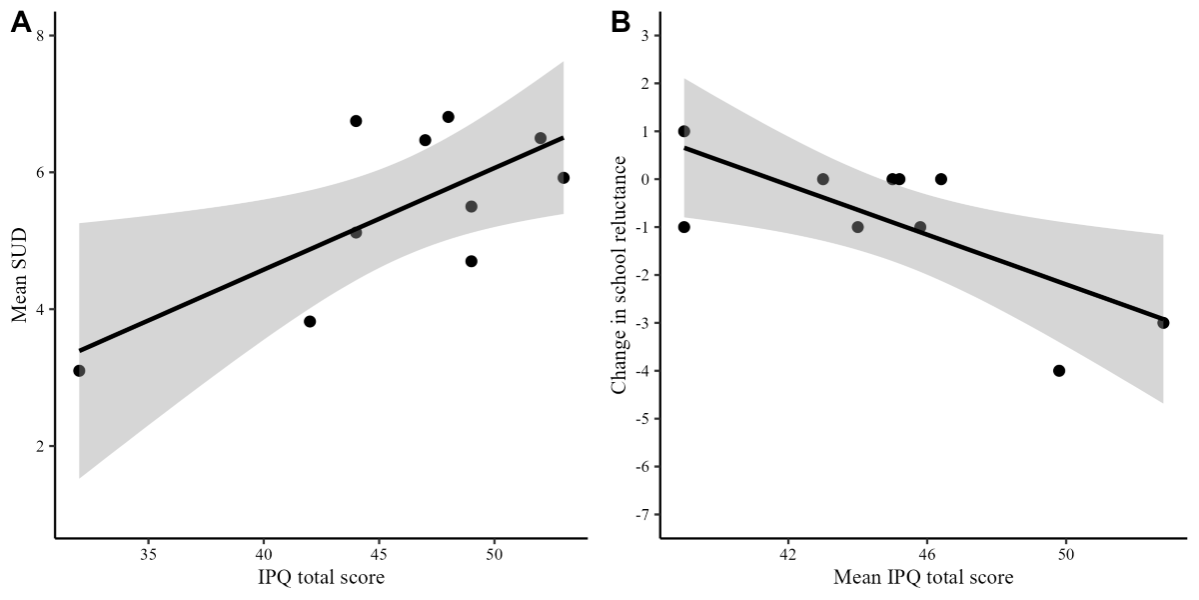
Association between presence and Subjective Units of Distress (state anxiety) in the first session and between mean presence across sessions and pre to post change in school reluctance (N=10). The graph represents the regression line with standard error. (B) Change was calculated as a Post-pre-difference , so that negative values represent a reduction from pre to post-VRET. IPQ: Igroup Presence Questionnaire; SUD: Subjective Units of Distress; VRET: virtual reality exposure therapy.

A significant correlation between the IPQ total score and mean SUD during the first session was observed (*r*=0.70; *P*=.01; 95% CI 0.24-1.00), suggesting that higher levels of presence were associated with higher levels of state anxiety (Figure 3A), but not with HR (*r*=–0.13; *P*=.58; 95% CI –0.86 to 1.00).

In addition, there was a negative correlation between the mean IPQ score across all five sessions and the post- and predifference scores in school reluctance (*r*=–0.73; *P*=.009; *P*_corr._=.03; 95% CI –1.00 to –0.29), indicating that higher levels of presence were associated with greater reductions in school reluctance (Figure 3B). However, no correlation was found between mean presence across sessions and the post- and predifferences in test anxiety (*r*=–0.19; *P*=.29; *P*_corr_.=.42; 95% CI –1.00 to 0.40) and general anxiety (ρ=–0.29; *P*=.21; *P*_corr._=.42; 95% CI –0.78 to 0.42).

## Discussion

### Principal Findings

This study examined a novel VRET intervention to reduce school anxiety in adolescents. While there is consistent evidence for the efficacy of VRET for the treatment of anxiety disorders in adult populations [33,40,49], it is currently unclear whether VRET may be also effective in adolescents. The VR scenario elicited state anxiety consistently across participants, particularly in the first session with a large effect size. VRET was associated with a reduction in state anxiety and social anxiety symptoms, as well as a trend toward a decrease in trait school anxiety, suggesting the feasibility and potential effectiveness of VRET in the treatment of school and social anxiety symptoms in adolescents. Autonomic arousal did not change. Presence during VR exposure was associated with state anxiety and treatment response in a subscale of the AFS measuring school reluctance.

The strongest state anxiety ratings were reported during the tasks inside the classroom, suggesting that anxiety was not only triggered by the general laboratory and VR setting but specifically by anxiety-relevant stimuli and tasks within the scenario. This finding aligns with previous research demonstrating that virtual social-evaluative situations can evoke anxiety in both adults [40,74-77] and youth [44].

The effects of VRET on HR were less clear potentially due to the small sample size (n=4). However, HR was elevated inside the classroom, especially in the first session. In addition, although not statistically tested, throughout the whole exposure, HR was higher than in adolescents at rest reported by other studies [78,79] suggesting that the VR scenario also elicited autonomic arousal consistent with previous findings [47,74,76,77,80,81]. Autonomic arousal may have been less specifically linked to the anxiety-relevant tasks than self-reported anxiety, as youth with SAD are discussed to show blunted autonomic reactivity [79,82-84].

VRET was associated with a reduction in state anxiety across all participants (η^2^=0.74), potentially caused by mechanisms of inhibitory learning and/or habituation in the specific situation. Moreover, we also observed a pre- to post reduction in social anxiety and a trend for a decrease in trait school anxiety. These findings are consistent with previous research on VRET for SAD and public speaking anxiety in adults [40,41,85,86], as well as with studies examining VRET in youth on public speaking and school anxiety [47,48]. These findings suggest that the VRET approach may be used to reduce state and potentially also for trait anxiety in adolescents.

However, despite large effects on state anxiety, the effect sizes and clinical response rates related to trait school anxiety in this study were below the typical outcomes of CBT and exposure therapy for SAD and school anxiety [23,42,87,88]. Moreover, most participants did not show reliable changes in trait school anxiety.

Several factors may account for these findings. First, exposure therapy for SAD and school refusal is mostly not examined as a stand-alone treatment, but rather in combination with other cognitive-behavioral methods such as cognitive restructuring [23,40,87,89]. In SAD and school anxiety, the cognitive component may play a greater role than in specific phobias, partly because of interpretation biases, indicating the need for cognitive interventions alongside exposure therapy [5,90,91]. Second, our VRET involved comparatively few sessions [87,89], which may be relevant since the number of treatment sessions may be a moderator of pre- to postanxiety reduction in CBT interventions [88]. Additionally, we included only one scenario, although multiple contexts and various stimuli are recommended to enhance therapeutic effects [51,89,92,93]. Furthermore, additional factors should be addressed, such as bullying and peer relationships, as these are considered potential contributors to the maintenance of school anxiety and school refusal [6,7,16]. Thus, incorporating social skills training might enhance treatment outcomes [88]. Finally, the majority of participants had comorbid depression, which may have negatively affected the treatment response [94,95].

VRET in our study was not associated with a reduction in autonomic arousal. These data are consistent with a previous study on CBT for children with SAD [82]. Accordingly, it is possible that autonomic changes require longer time scales to occur following psychological interventions [82,96], especially in anxiety disorders that are more complex than specific phobias. Moreover, the task demands of the VR scenario may have contributed to the absence of HR reductions [82] as social-evaluative tasks and cognitive load can elicit autonomic arousal even in healthy individuals [97-99].

The analyses of presence yielded a strong positive correlation with state anxiety in the first session. While this finding is consistent with previous research, the causality and direction of this effect, currently discussed to be bidirectional, should be further explored [55,59,80,100]. In addition, our results indicate a relationship between presence and treatment response, in particular school reluctance, aligning with prior research on VRET for public speaking anxiety [55,60]. It should be noted, however, that no correlation was found with the other subscales of trait school anxiety. Given the heterogeneous literature [47,59], this emphasizes the need for further research on presence and its relevance to VRET’s therapeutic success.

### Limitations

The sample size of this pilot study was small (n=10). In addition, the results derive from uncorrected *P* values and should be interpreted with caution. Moreover, cognitive symptoms and autonomic responses in social-evaluative situations can also depend on factors such as age and type of sample [98,101]. Hence, the findings may not generalize to other groups with school anxiety, especially children and individuals without SAD. Moreover, the pilot study did not include a control or comparison group. Therefore, we cannot separate the effects of VRET from other factors, such as spontaneous remission, the concurrent therapy also targeting SAD, school visits, and nonspecific therapeutic factors due to contact with the experimenter.

Another limitation pertains to the presence of the experimenter during VR exposure, which could have contributed to the anxiety response. In addition, VRET was not conducted by trained therapists, which might have reduced its effectiveness. Nonetheless, previous studies also employing nonexpert therapists have observed reductions in anxiety as well [46,102] with lay therapists potentially being a highly sought-after resource to bridge the current gap between demand and mental health care. Furthermore, while the ability to move may have increased the presence during the VR exposure, it limits the autonomic results, as HR can be influenced by movement and position changes [103].

### Conclusions

In summary, this pilot study offers preliminary evidence regarding the feasibility and potential effectiveness of a newly developed VRET for adolescents with school anxiety related to SAD, suggesting that VRET may constitute an effective treatment component for symptoms of anxiety in adolescence. The VR scenario successfully elicited self-reported state anxiety and autonomic arousal. Importantly, reductions in self-reported state anxiety and trait social anxiety, as well as a trend for a decrease in trait school anxiety were observed after five exposure sessions. Incorporation of a greater number of virtual scenarios and cognitive elements into VRET may further improve treatment outcomes. Future studies and RCTs should address the effectiveness of VRET for adolescents with school anxiety in large samples that include a waiting list group, active psychological placebo, or exposure in vivo to better examine and compare the efficacy of the VRET approach.

## Appendix

Multimedia Appendix 1 Study protocols.

Multimedia Appendix 2 Results of relevant post hoc pairwise comparisons of state anxiety and autonomic parameters.

Multimedia Appendix 3 Individual trajectories of state anxiety and autonomic parameters.

Multimedia Appendix 4 Percentage change and RCI (reliable change index) in trait school anxiety and social anxiety from pre to post VRET (virtual reality exposure therapy).

## Abbreviations

AFS: Angstfragebogen für Schüler (English: Anxiety Questionnaire for Pupils)
CBT: cognitive behavioral therapy
DISYPS-III (SBB-ANG): Diagnostic System for Mental Disorders in Children and Adolescents in German (anxiety disorder section, self-report)
HR: heart rate
ICD-10: International Statistical Classification of Diseases, Tenth Revision
IPQ: Igroup Presence Questionnaire
RCI: reliable change index
SAD: social anxiety disorder
SUD: subjective units of distress
VR: virtual reality
VRET: virtual reality exposure therapy
