# Supplementary material for: Virtual Reality Exposure Therapy for Reducing School Anxiety in Adolescents: Pilot Study
Source: JMIR Ment Health. 2024 Nov 5;11:e56235. doi: 10.2196/56235 (PMC11576610; doi:10.2196/56235)
Supplement: Multimedia Appendix 1 [file mental_v11i1e56235_app1.docx]

*English short version (summary) see below*

**Semi-structured Guideline for Introduction to Exposure**

*1. Sitzung (nach Aufklärung)*

- Angst
  - Angst kennt jeder, sie ist normal, notwendig und harmlos. Denn sie ist eine **Schutzfunktion**, sie hilft uns aufmerksam zu sein, Gefahren zu erkennen. Sie ist eine Reaktion auf ein Gefühl in unserem Körper oder auf eine Situation außerhalb unseres Körpers. Wir möchten nur die Angst loswerden, die uns nicht hilft, weil sie da ist, obwohl keine reale Gefahr besteht.
  - **Ebenen**: Zur Angst gehören Gedanken, Körpersymptome und Verhalten.
  - Was spürst du in deinem Köper, wenn du Angst hast?
  - Was geht dir durch den Kopf, wenn du ängstlich bist?
  - Was tust du, wenn du Angst hast?
  - Wo im Körper spürst du die Angst?
    - Erklärung der Nutzung des **Pulsgerätes**: Um genau dies auch innerhalb dieser Studie zu erfassen, nutzen wir dieses Pulsgerät hier. Daher möchte ich, dass du dieses gleich umlegst, sodass wir auch wissen, was unser Szenario mit deinem Puls macht.
- Konfrontationsrational, Kognitive Vorbereitung
  - Wir haben ja schon kurz über Expositionen, also die Konfrontation mit der angstauslösenden Situation gesprochen. Weißt du noch, was das **Ziel bzw. deine Aufgabe** ist? (Angst aushalten, bis es zu einer Reduktion der Angst kommt)
  - Häufig machen wir ja Dinge, um die **Angst zu reduzieren**, wenn wir das Gefühl haben, sie nicht länger aushalten zu können. Wie verhältst du dich in der Regel, wenn die Angst steigt?
  - Ohne Vermeidung: Vielleicht kennst du es, dass man die **Erwartung** hat, dass die Angst bis ins Unermessliche steigen könnte. Was glaubst du, wie stark kann die Angst steigen?
  - Ein wichtiger Faktor bei der Aufrechterhaltung der Angst, ist, dass man **vermeidet**, also Dinge macht, um die Angst zu reduzieren oder sich gar nicht erst der Situation stellt. Dadurch lässt es sich gar nicht erleben, dass die Anspannung abnimmt und lernen, dass man mit der Angst umgehen kann, dass sie **auszuhalten und zu bewältigen** ist.
  - Worauf muss ich also bei der Übung bei dir achten? Gibt es **Vermeidungsstrategien oder Hilfsmittel**, die du nutzt, die die Angst aber langfristig aufrechterhalten? Dann kann ich dich unterstützen, dich komplett der Angst zu stellen.
- Und damit du das ganze häufiger erleben kannst und vielleicht auch pro Konfrontation bereits weniger Angst hast, machen wir das ganze **fünf Mal**.
- Du wirst immer dieselben **Aufgaben** bekommen.
- Alle **Instruktionen** erhältst du über die Kopfhörer. Ich werde dir aber nur einen direkt aufs Ohr setzen, damit du auch mich noch hören kannst. (Instruktionen sagen immer, was zu tun ist. Sonst Zeit zum Umschauen etc.).
- Zuerst Eingewöhnung vor dem Schulgelände. Du wirst dann durch deine Schule laufen können und hast anschließend die Aufgabe, dein Klassenzimmer aufzusuchen und dich an deinen Platz zu setzen. Ich werde dir dafür hier im Raum einen **Stuhl** hinstellen, bitte setze dich erst, wenn ich hier in diesem Raum sage, dass du dich setzen kannst. Danach wirst du aufgefordert, dich vor die Klasse zu stellen, dich vorzustellen, ein paar Rechenaufgaben zu lösen und von einem Erlebnis zu erzählen, das du in den letzten Wochen erlebt hast. Die Fragen werden sich vielleicht etwas wiederholen, wir wollen das ja so lange machen, bis sich deine Angst reduziert hat.
- Falls du merkst, dass die **Angst zu stark** ist, sag mir bitte Bescheid. Dann gehen wir einen Schritt zurück und du bleibst erstmal in der Situation. Das ist auch vollkommen ok. Es geht ja darum, dass du deine Angst aushältst, das ist das Wichtige, die Situationen können wir auch im Verlauf noch steigern.
- Gut, dann würde ich dich jetzt erstmal bitten, folgende **Fragebögen** auszufüllen.
- Frage nach **Toilette**
- Was ist denn deine schlimmste **Befürchtung**, was passieren wird? Auf einer Skala von 0 bis 100, wie stark glaubst du das?
- **SUD:** Während der Zeit in der virtuellen Realität wirst du regelmäßig nach deiner Angst gefragt. Dafür nutzen wir diese Skala (SUD Bild zeigen). Diese geht von 0 entspannt keine Angst, bis 10 größte Angst aller Zeiten. (Beispiele für andere Zahlen geben, siehe SUD). Wie sieht‘s denn gerade bei dir aus?
- Erklärung Controller (rechts springen, links laufen, greifen; im Klassenzimmer nicht mehr springen)
- Stuhl erneut betonen
- Pulsgerät anlegen lassen
- Anlegen VR
- Ende
  - Ablegen, erstmal wieder ankommen lassen im Raum
  - Wie geht’s dir? Wie fühlst du dich?
  - Ist die schlimmste Befürchtung, die du vorher genannt hattest, eingetreten?
  - Wie weißt du das? Wenn ja: Wie bist du damit umgegangen? Wenn nein: Wie erklärst du dir das?
  - Verstärkung, sich der Situation ausgesetzt zu haben
  - Nun möchte ich dich noch einmal bitten, Fragebögen auszufüllen.

*2.-5. Sitzung:*

Beginn

- Du kennst das ganze hier ja jetzt schon. Weißt du noch, was das **Ziel bzw. deine Aufgabe** ist?
- Worauf muss ich also bei der Übung bei dir achten? Gibt es **Vermeidungsstrategien oder Hilfsmittel**, die du nutzt, die die Angst aber langfristig aufrechterhalten? Dann kann ich dich unterstützen, dich komplett der Angst zu stellen.
- Die **Aufgaben** kennst du nun ja auch schon, kannst du dich noch erinnern?
- Falls du merkst, dass die **Angst zu stark** ist, sag mir bitte Bescheid. Dann gehen wir einen Schritt zurück und du bleibst erstmal in der Situation. Das ist auch vollkommen ok, es geht ja darum, dass du deine Angst aushältst, das ist das Wichtige, die Situationen können wir auch im Verlauf noch steigern.
- Gut, dann würde ich dich jetzt erstmal bitten, folgende **Fragebögen** auszufüllen.
- Frage nach **Toilette**
- Was ist denn deine schlimmste **Befürchtung**, was passieren wird? Auf einer Skala von 0 bis 100, wie stark glaubst du das?
- **SUD**: Während der Zeit in der virtuellen Realität wirst du regelmäßig nach deiner Angst gefragt. Erinnerst du dich an die Skala (SUD zeigen)? Wie sieht‘s denn gerade bei dir aus?
- Erklärung Controller (rechts springen, links laufen, greifen)
- Stuhl erneut betonen
- Pulsgerät anlegen lassen
- Anlegen VR

Ende

- Ablegen, erstmal wieder ankommen lassen im Raum
- Wie geht’s dir? Wie fühlst du dich?
- Ist die schlimmste Befürchtung, die du vorher genannt hattest, eingetreten?
- Wie weißt du das? Wenn ja: Wie bist du damit umgegangen? Wenn nein: Wie erklärst du dir das?
- Verstärkung, sich der Situation ausgesetzt zu haben
- Nun möchte ich dich noch einmal bitten, Fragebögen auszufüllen.

**Semi-structured Guideline for Introduction to Exposure**

*1. Session*

- Psychoeducation about fear: protective function, levels (thoughts, body symptoms and behavior)
- Explanation of the use of the pulse device
- Cognitive preparation: Psychoeducation about rationale of exposure; the participants’ specific avoidance and safety behavior and its relevance to the maintenance of anxiety; expected course of anxiety with and without avoidance
- Explanation of procedure: 5 exposure sessions with the same procedure and tasks, explanation of the phases
- Introduction to VR equipment: headphones, controller
- Questionnaires: AFS (anxiety questionnaire for pupils), DISYPS (diagnostic system for mental disorders in children and adolescents in German)
- Verbal assessment of worst expectancy that could occur during exposure and likelihood of its actual occurrence on a scale from 0 to 100
- Explanation of SUD (subjective units of distress) assessment with a visual scale
- Putting on the pulse device and VR equipment
- Virtual exposure session
- End:
  - Welcoming back
  - “Did the worst fear you mentioned before come true? How do you know? If yes: How did you deal with it? If no: How do you explain this?”
  - Reinforcement of having been exposed to the situation
  - Questionnaire: IPQ

*2^nd^ – 5^th^ Session*

- Start
  - Short repetition of the goal and the task during exposure, avoidance and safety behavior as well as procedure
  - Questionnaires: not relevant to this paper
  - Assessment of negative expectancy
  - Putting on the pulse device, then putting on VR
- End: as in session 1

**Guideline for VR Exposure Sessions**

- The experimenter guided the participants through the exposure sessions using pre-recorded instructional sentences and sentences spoken by the teacher.
- From session two onwards, anxiety was increased by reactions of classmates: glances from classmates, applauding or laughing at the participant.

1. General Sentences that were regularly used
   - SUD: *On the familiar scale of 0 (relaxed, no anxiety) to 10 (most anxious ever), how high is your anxiety right now?*
   - Body: *What do you feel in your body?*
   - Stopping avoidance/safety behaviors: *Are you doing anything to reduce your anxiety right now?*
     - *It's good that you're telling me this. What should you be doing right now to make the exercise successful?*
     - *Great*
   - Anxiety too high: *Okay, anxiety is high right now and you rather want to stop. Stay with the fear for a moment, allow it and don't push it back. You're doing great!*
     - *I'm offering that we go back a little bit.*
2. Acclimatization
   - Familiarization to VR on street, 3 minutes
   - *First, please find your way around a little first. To do this, walk around a bit, pick up the ball with the controllers and take a good look at everything.*
   - Assessment of SUD and body symptoms
   - Transition to next phase: *If you are ready, you can now enter the school grounds. Press the button to open the door.*
3. Exploration
   - *Now you can have a look at your school. First move freely through the building or the schoolyard.* (two – five minutes)
   - *I would now like you to walk to your classroom.*
   - Assessment of SUD in school corridor
   - Transition to next phase: *When you're ready, you're welcome to go in.*
   - In classroom: *Do you see your seat? Please sit there.* Put down chair
4. Class
   - School bell (= class start/stop)
   - After 1 min SUD
   - Pupils looking at participant from session 2 onwards (letting them turn away and back again)
   - After 2 minutes: *Please come to the front of the class. I have a few questions for you.*
   - *Please stand so the class can see you?; Are you ready?*
   - SUD and body symptoms
5. Introduction
   - *Since you are new to the class, I would like you to introduce yourself.*
   - Supporting questions: *What is your name? How old are you? Where do you live? Where are you from? What is your favorite subject at school? What do you like about it? Which school subject do you not like at all? What is your favorite hobby? What do you like about it?*
   - Teacher’s reactions: *Mmmh, ok. Thank you.*
   - SUD
6. Math (two – three tasks)
   - *I now have one/two math problems for you and I want you to answer them out loud in front of everyone. Got it?* - *Ok, here we go.*
   - Tasks: *What is … 28+12; 8*6; 34 m in cm; 72 : 4; 5² = 25; 11*11; 100 - 6 = 600; ½ of 70; 265 + 35; 280+ 36; 5 + 7*9; 5 600 : 8; 3 + 4*8; What is the middle number between 2.1 and 3.6? A jacket costs 40€. In the winter sale it is reduced by 25%, how much does it still cost?*
   - SUD and body symptoms
7. Event
   - Task: *I would like you to tell us about something you have experienced in the last few weeks. What kind of experience can you think of?*
   - Supporting questions: *What did you do? That sounds good. Tell us a bit about it! Where was it? Who was there? What did you like most about it? When was that? And what else did you do? What else can you tell us about it? What did you do afterwards? What didn't you like so much?*
   - Teacher’s reactions: *That sounds nice. That sounds exciting. It sounds like an exciting experience. I see. Thank you.*
   - SUD and body symptoms: first time after 2-3 questions from teacher, then every 4 minutes
8. Mixed/ Coming to an end
   - Continuing tasks in front of the class until 40% reduction of fear: if necessary, tasks of event and math were mixed.
   - Only if necessary, experimenter asked a question (holiday, favorite book, film etc.) 🡪 going back to only letting the teacher speak as soon as possible
9. End
   - *Thank you for your detailed story. You can now sit down again."*
   - Sitting in the classroom, putting the chair back down.
   - School bell (lesson start/stop)
   - After 1 min SUD
   - *You have done it. I will finish now. It will be dark for a short time.*
