# Supplementary material for: Virtual Reality Exposure Therapy for Reducing School Anxiety in Adolescents: Pilot Study
Source: JMIR Ment Health. 2024 Nov 5;11:e56235. doi: 10.2196/56235 (PMC11576610; doi:10.2196/56235)
Supplement: Multimedia Appendix 2 [file mental_v11i1e56235_app2.docx]

Table S1. Post Hoc Pairwise Comparisons of Within-Sessions Main Effects of State Anxiety and Heart Rate.

| Comparison | | SUD | | | | | | HR | | | | |
| --- | --- | --- | --- | --- | --- | --- | --- | --- | --- | --- | --- | --- |
|  |  | *t*(19) | |  | *p* | adj. *p* | | *t*(7) | | *p* | adj. *p* | |
| Class | Acclimatization | 6 | .16 | **<** | **.001** |  | **.002** | -2 | .64 | **.033** |  | .233 |
|  | Exploration | 3 | .81 |  | **.001** |  | **.012** | -2 | .39 | **.048** |  | .288 |
|  | Introduction | -2 | .34 |  | **.044** |  | .920 | -4 | .30 | **.004** |  | .054 |
|  | Math | -3 | .01 |  | **.007** |  | .051 | -7 | .09 | **<.001** |  | .**004** |
|  | Event | -2 | .24 |  | **.038** |  | .190 | -3 | .72 | **.007** |  | .092 |
|  | End | 4 | .30 | **<** | **.001** |  | **.005** | 1 | .86 | .105 |  | .525 |
| Introduction | Acclimatization | 5 | .67 | **<** | **.001** | **<** | **.001** | 4 | .38 | **.003** |  | .052 |
|  | Exploration | 4 | .11 | **<** | **.001** |  | **.007** | 3 | .76 | **.007** |  | .092 |
|  | Math | 2 | .16 |  | **.040** | 0 | .191 | -1 | .37 | .213 |  | .852 |
|  | Event | -0 | .506 |  | .691 |  | .806 | -0 | .95 | .375 | 1 | .000 |
|  | End | 5 | .52 | **<** | **.001** | **<** | **.001** | 5 | .12 | **.001** |  | **.026** |
| Math | Acclimatization | 5 | .90 | **<** | **.001** | **<** | **.001** | 4 | .94 | **.002** |  | **.030** |
|  | Exploration | 4 | .45 | **<** | **.001** |  | **.004** | 3 | .92 | **.006** |  | .080 |
|  | Event | 2 | .32 |  | **.032** |  | .191 | 0 | .40 | .699 | 1 | .000 |
|  | End | 6 | .77 | **<** | **.001** | **<** | **.001** | 4 | .93 | **.002** |  | **.030** |
| Event | Acclimatization | 5 | .18 | **<** | **.001** | **<** | **.001** | 3 | .17 | **.016** |  | .158 |
|  | Exploration | 3 | .54 |  | **.002** |  | **.018** | 2 | .78 | **.028** |  | .220 |
|  | End | 6 | .10 | **<** | **.001** | **<** | **.001** | 7 | .41 | **<.001** |  | **.003** |
| Acclimatization | Exploration | -3 | .83 |  | **.001** |  | **.012** | -0 | .48 | .644 | 1 | .000 |
|  | End | -0. | 856 |  | .403 |  | .806 | 3 | .32 | **.013** |  | .141 |
| Exploration | End | 1. | 40 |  | .179 |  | .537 | 3 | .13 | **.017** |  | .158 |

*Note.* SUD = Subjective Units of Distress (state anxiety); HR = heart rate; Bonferroni-Holm correction was applied for adjusted *p*-values. Marked are *p*-values ≤ .05.

Table S2. Post Hoc Pairwise Comparisons Between Final and First Sessions of State Anxiety.

| Comparison | SUD | | | | | |
| --- | --- | --- | --- | --- | --- | --- |
|  | *t*(10) | |  | *p* | adj. *p* | |
| Acclimatization | -3 | .69 | **<** | **.001** |  | **.002** |
| Class | -5 | .06 | **<** | **.001** | **<** | **.001** |
| Exploration | -3 | .27 |  | **.01** |  | **.01** |
| Introduction | -6 | .09 | **<** | **.001** | **<** | **.001** |
| Math | -4 | .62 |  | **.001** |  | **.001** |
| Event | -4 | .54 |  | **.001** |  | **.001** |
| End | -2 | .32 |  | **.046** |  | **.046** |
| Final – First Session, *t*(69) | -10 | .48 | **<** | **.001** | < | **.001** |

*Note.* SUD = Subjective Units of Distress (state anxiety); Bonferroni-Holm correction was applied for adjusted *p*-values. Marked are *p*-values ≤ .05.

Table S3. Post Hoc Pairwise Comparisons of State Anxiety Within the First and the Final Session.

| Session | Comparison | | SUD | | | | | |
| --- | --- | --- | --- | --- | --- | --- | --- | --- |
|  |  |  | *t*(9) | | *p* | | adj. *p* | |
| First Session | Class | Acclimatization | 5 | .16 | **<** | **.001** |  | **.01** |
|  |  | Exploration | 3 | .63 |  | **.005** |  | .054 |
|  |  | Introduction | -3 | .21 |  | **.011** |  | .086 |
|  |  | Math | -4 | .73 |  | **.001** |  | **.014** |
|  |  | Event | -2 | .46 |  | **.036** |  | .220 |
|  |  | End | 3 | .42 |  | **.008** |  | .069 |
|  | Introduction | Acclimatization | 5 | .51 | **<** | **.001** |  | **.006** |
|  |  | Exploration | 3 | .95 |  | **.003** |  | .040 |
|  |  | Math | -1 | .81 |  | .104 |  | .520 |
|  |  | Event | -0 | .41 |  | .691 | 1 | .000 |
|  |  | End | 4 | .82 | **<** | **.001** |  | **.013** |
|  | Math | Acclimatization | 6 | .78 | **<** | **.001** |  | **.002** |
|  |  | Exploration | 5 | .08 | **<** | **.001** |  | **.010** |
|  |  | Event | 1 | .78 |  | .109 |  | .520 |
|  |  | End | 8 | .12 | **<** | **.001** | **<** | **.001** |
|  | Event | Acclimatization | 5 | .65 | **<** | **.001** |  | **.006** |
|  |  | Exploration | 3 | .88 |  | **.004** |  | **.041** |
|  |  | End | 8 | .20 | **<** | **.001** | **<** | **.001** |
|  | Acclimatization | Exploration | -2 | .55 |  | **.031** |  | .220 |
|  |  | End | -0 | .57 |  | .583 | 1 | .000 |
|  | End | Exploration | -1 | .56 |  | .154 | 1 | .000 |
| Final Session | Class | Acclimatization | 4 | .12 |  | **.003** |  | .055 |
|  |  | Exploration | 2 | .33 |  | **.045** |  | .935 |
|  |  | Introduction | -1 | .34 |  | .215 | 1 | .000 |
|  |  | Math | -1 | .46 |  | .177 | 1 | .000 |
|  |  | Event | -0 | .97 |  | .357 | 1 | .000 |
|  |  | End | 2 | .87 |  | **.019** |  | .393 |
|  | Introduction | Acclimatization | 2 | .95 |  | **.016** |  | .290 |
|  |  | Exploration | 2 | .06 |  | .070 |  | .767 |
|  |  | Math | -1 | .15 |  | .279 | 1 | .000 |
|  |  | Event | -0 | .28 |  | .785 | 1 | .000 |
|  |  | End | 3 | .30 |  | **.009** |  | .183 |
|  | Math | Acclimatization | 2 | .75 |  | **.022** |  | .358 |
|  |  | Exploration | 1 | .88 |  | .093 |  | .932 |
|  |  | Event | 1 | .54 |  | .157 | 1 | .000 |
|  |  | End | 3 | .26 |  | **.010** |  | .186 |
|  | Event | Acclimatization | 2 | .42 |  | **.039** |  | .502 |
|  |  | Exploration | 1 | .43 |  | .186 | 1 | .000 |
|  |  | End | 2 | .68 |  | **.025** |  | .358 |
|  | Acclimatization | Exploration | -2 | .75 |  | **.022** |  | 358 |
|  |  | End | -0 | .67 |  | .521 | 1 | .000 |
|  | End | Exploration | -1 | .56 |  | .154 | 1 | .000 |

*Note.* SUD = Subjective Units of Distress (state anxiety); Bonferroni-Holm correction was applied for adjusted *p*-values. Marked are *p*-values ≤ .05.
