# Supplementary material for: Virtual Reality Exposure Therapy for Reducing School Anxiety in Adolescents: Pilot Study
Source: JMIR Ment Health. 2024 Nov 5;11:e56235. doi: 10.2196/56235 (PMC11576610; doi:10.2196/56235)
Supplement: Multimedia Appendix 3 [file mental_v11i1e56235_app3.docx]

Figure S1. Trajectories of State Anxiety Within the First (A) and the Final (B) Session.

*
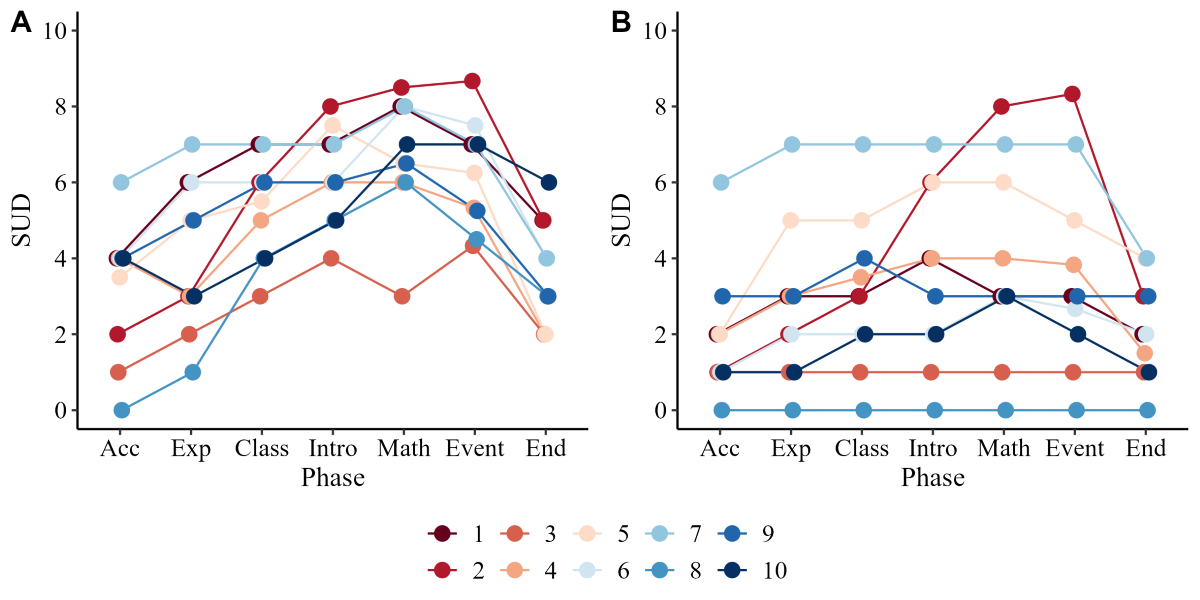
*

*Note*. Acc = Acclimatization, Exp = Exploration, Intro = Introduction, SUD = Subjective Units of Distress (state anxiety).

Figure S2. Trajectories of Heart Rate Within the First (A) and Final (B) Session.

*
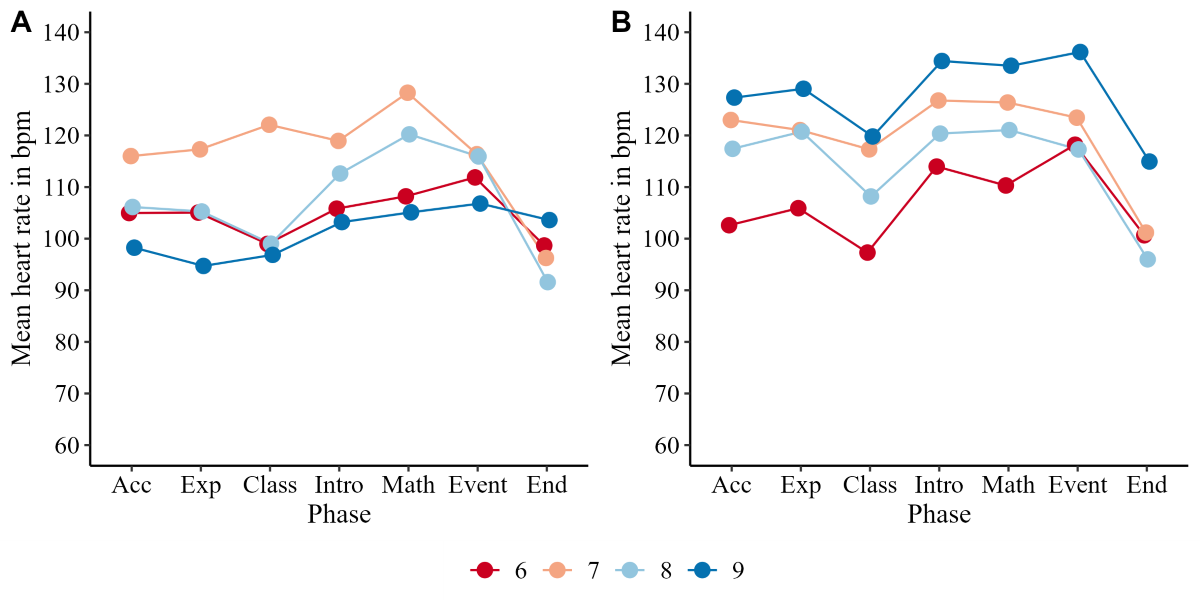
*

*Note*. Acc = Acclimatization, Exp = Exploration, Intro = Introduction.
