# Supplementary material for: Virtual Reality Exposure Therapy for Reducing School Anxiety in Adolescents: Pilot Study
Source: JMIR Ment Health. 2024 Nov 5;11:e56235. doi: 10.2196/56235 (PMC11576610; doi:10.2196/56235)
Supplement: Multimedia Appendix 4 [file mental_v11i1e56235_app4.docx]

| PCP | Test Anxiety | | | | General Anxiety | | | | School Reluctance | | | | Social Anxiety | |
| --- | --- | --- | --- | --- | --- | --- | --- | --- | --- | --- | --- | --- | --- | --- |
|  | % Change | | RCI | | % Change | | RCI | | % Change | | RCI | | % Change | |
| 1 | -15 | .4 | -0 | .67 | **-30** | **.8** | -1 | .32 | **-50** | **.0** | -1 | .67 | **-33** | **.3** |
| 2 | 0 | .0 | 0 | .00 | -7 | .7 | -0 | .33 | 0 | .0 | 0 | .00 | **-31** | **.3** |
| 3 | -7 | .7 | -0 | .34 | -8 | .3 | -0 | .33 | -12 | .5 | -0 | .56 | -6 | .3 |
| 4 | -6 | .7 | -0 | .34 | **-42** | **.9** | **-1** | **.97** | **-57** | **.1** | **-2** | **.23** | 0 | .0 |
| 5 | 0 | .0 | 0 | .00 | 0 | .0 | 0 | .00 | -11 | .1 | -0 | .56 | 0 | .0 |
| 6 | 16 | .7 | 0 | .67 | 0 | .0 | 0 | .00 | -11 | .1 | -0 | .56 | -28 | .6 |
| 7 | -13 | .3 | -0 | .67 | 18 | .2 | 0 | .66 | 0 | .0 | 0 | .00 | -5 | .3 |
| 8 | 0 | .0 | 0 | .00 | -7 | .1 | -0 | .33 | 11 | .1 | 0 | .56 | -5 | .6 |
| 9 | **-30** | **.0** | -1 | .01 | 0 | .0 | 0 | .00 | 0 | .0 | 0 | .00 | -17 | .7 |
| 10 | 0 | .0 | 0 | .00 | 0 | .0 | 0 | .00 | 0 | .0 | 0 | .00 | 7 | .7 |

*Note*. PCP = participant; Change was calculated as post-pre differences with negative values representing a reduction from pre to post VRET; RCI = reliable change index; Marked are percentage changes meeting the response criteria of > |30| or RCI > |1.96|; Social anxiety was measured with the Diagnostic System for Mental Disorders in Children and Adolescents in German (DISYPS-III-SBB-ANG), Test Anxiety, General Anxiety and School Reluctance were measured with the Anxiety Questionnaire for Pupils (AFS).
